# *Drosophila suzukii* population response to environment and management strategies

**DOI:** 10.1007/s10340-016-0757-4

**Published:** 2016-04-01

**Authors:** Nik G. Wiman, Daniel T. Dalton, Gianfranco Anfora, Antonio Biondi, Joanna C. Chiu, Kent M. Daane, Beverly Gerdeman, Angela Gottardello, Kelly A. Hamby, Rufus Isaacs, Alberto Grassi, Claudio Ioriatti, Jana C. Lee, Betsey Miller, M. Valerio Rossi Stacconi, Peter W. Shearer, Lynell Tanigoshi, Xingeng Wang, Vaughn M. Walton

**Affiliations:** Department of Horticulture, Oregon State University, 4017 Ag and Life Sciences Bldg., Corvallis, OR 97331 USA; Research and Innovation Centre and Technology Transfer Centre, Fondazione Edmund Mach, Via E. Mach, 1, 38010 San Michele all’Adige, TN Italy; Department of Agriculture, Food and Environment, University of Catania, Via Santa Sofia, 100, 95123 Catania, Italy; Department of Entomology and Nematology, University of California, Davis, CA 95616 USA; Department of Environmental Science, Policy and Management, University of California, Berkeley, CA 94720-3114 USA; Department of Entomology, Mount Vernon Northwestern Washington Research and Extension Center, Washington State University, Mount Vernon, WA 98273-4768 USA; Department of Entomology, University of Maryland, 4112 Plant Sciences Building, College Park, MD USA; Department of Entomology, Michigan State University, East Lansing, MI 48824 USA; USDA-ARS Horticultural Crops Research Unit, 3420 NW Orchard Ave., Corvallis, OR 97330 USA; Mid-Columbia Agricultural Research and Extension Center, Oregon State University, Hood River, OR 97031 USA; Department of Horticulture, Oregon State University, 4105C ALS, Corvallis, OR 97331 USA

**Keywords:** DD, Temperature, Fecundity, Survival, Population dynamics, Pesticide, Spotted wing *Drosophila*

## Abstract

*Drosophila suzukii* causes economic damage to berry and stone fruit worldwide. Laboratory-generated datasets were standardized and combined on the basis of degree days (DD), using Gompertz and Cauchy curves for survival and reproduction. Eggs transitioned to larvae at 20.3 DD; larvae to pupae at 118.1 DD; and pupae to adults at 200 DD. All adults are expected to have died at 610 DD. Oviposition initiates at 210 DD and gradually increases to a maximum of 15 eggs per DD at 410 DD and subsequently decreases to zero at 610 DD. These data were used as the basis for a DD cohort-level population model. Laboratory survival under extreme temperatures when DD did not accumulate was described by a Gompertz curve based on calendar days. We determined that the initiation of the reproductive period of late dormant field-collected female *D. suzukii* ranged from 50 to 800 DD from January 1. This suggests that *D. suzukii* females can reproduce early in the season and are probably limited by availability of early host plants. Finally, we used the DD population model to examine hypothetical stage-specific mortality effects of IPM practices from insecticides and parasitoids at the field level. We found that adulticides applied during the early season will result in the largest comparative population decrease. It is clear from model outputs that parasitism levels comparable to those found in field studies may have a limited effect on population growth. Novel parasitoid guilds could therefore be improved and would be valuable for IPM of *D. suzukii*.

## Key message

- *Drosophila suzukii*, spotted wing drosophila, is a serious pest of small fruits and cherries.
- Key *D. suzukii* life-stage events over physiological time are described.
- *D. suzukii* survival rates are described under extreme environmental conditions.
- We demonstrate the impact of improving environmental conditions on *D. suzukii* reproductive potential.
- The relative population impacts of IPM actions targeting certain *D. suzukii* life stages are described.
- Future direction and refinements of population models are suggested.

## Introduction

*Drosophila suzukii* Matsumura (Diptera: Drosophilidae) causes economic damage to susceptible small and stone fruit in North America, Asia, and Europe (Asplen et al. 2015; Cini et al. 2012; Goodhue et al. 2011; Kanzawa 1939; Kawase et al. 2007; Lee et al. 2011; Walsh et al. 2011). Adult female flies oviposit in fruit and developing larvae render the high-value fresh fruit unmarketable (Murphy et al. 2015) and reduce processed fruit quality. Damage from *D. suzukii* in Western U.S.A. production areas may cause up to $500 million in annual losses assuming 30 % damage levels (Goodhue et al. 2011), and $207 million losses in Eastern U.S.A. production regions (NC Cooperative Extension 2015). Worldwide, the potential economic impacts due to *D. suzukii* damage are significant. In any integrated pest management (IPM) system, it is important to use multiple strategies to manage key pests. For *D. suzukii*, some of these strategies include monitoring, fruit sampling, and direct control methods (Walsh et al. 2011). For example, in Trento Province, Northern Italy, prior to the adoption of IPM, the potential losses to *D. suzukii* were about 13 % of the berry industry’s revenue, while after the implementation of an IPM strategy including mass trapping, field sanitation, and insecticide programs, the sum of losses and associated control costs decreased to about 7 % (De Ros et al. 2015).

The ability to describe, forecast, and more effectively manage damaging pest populations can benefit producers, extension agents, and practitioners (Almeida et al. 2010; Cianci et al. 2013; Focks et al. 1995; Jones and Wiman 2012). Phenology models based on accumulation of heat units or degree days (DD) (Baskerville and Emin 1969; Wilson and Barnett 1983) have become the standard method for determining when to treat crops for pests. These DD accumulation models can be used to predict important life history events based on pest development rates (Zalom and Goodell 1983). With phenology models, a specific life stage of a pest, such as adults, can be targeted for management, maximizing efficacy of insecticides. DD phenology models tend to work best for pests with a high level of synchrony and few, non-overlapping generations (Aghdam et al. 2009; Quesada-Moraga et al. 2012; Reissig et al. 1979; Rock and Shaffer 1983; Teixeira and Polavarapu 2001). Previous data have shown that *D. suzukii* moves through generations rapidly, and has high reproductive levels and overlapping generations (Emiljanowicz et al. 2014; Kinjo et al. 2014; Tochen et al. 2014). This suggests that limited benefits are to be gained from a traditional DD phenology model. However, insect population models can also be helpful to predict impending damage of agriculturally and medically important insect pest populations (Carey 1993, 2001; Wiman et al. 2014).

The major factors affecting population dynamics of *D. suzukii* include temperature, humidity (Shearer et al. 2016; Tochen et al. 2015), and the availability of essential food resources (Kimura 2004; Mitsui et al. 2006, 2007; Lee et al. 2015a). Although DD phenology models may have limited application for a pest such as *D. suzukii*, accumulation of heat units can play an important role in predicting population dynamics. Temperature-dependent developmental, survival, and reproductive data are available for all life stages of *D. suzukii* (Emiljanowicz et al. 2014; Hardin et al. 2015; Jaramillo et al. 2015; Tochen et al. 2014). Recent *D. suzukii* modeling has used a combination of mean temperature and calendar-based matrices (Asplen et al. 2015; Wiman et al. 2014). The two published demographic models for *D. suzukii* include a discrete-time stage-specific Leslie matrix model, which did not estimate transition between different life stages for *D. suzukii* (Wiman et al. 2014), and a physiologically based demographic model featuring distributed maturation time (Asplen et al. 2015). Asplen et al. (2015) used *D. suzukii* physiological data and included non-linear sub-models to capture temperature-dependent developmental rates and survivorship. Neither model takes into consideration winter survival, early-season reproductive potential, or host availability (Dalton et al. 2011; Lee et al. 2015a; Kaçar et al. 2016). However, attempts to model insect survival and fecundity using physiological time and matrices have been conducted successfully for other insects (Choi and Ryoo 2003).

Management strategies for *D. suzukii* include chemical (Beers et al. 2011; Bruck et al. 2011; Van Timmeren and Isaacs 2013; Wise et al. 2015), biological (Rossi Stacconi et al. 2013, 2015; Miller et al. 2015), and cultural (Lee et al. 2015b; Tochen et al. 2015) controls. Additional control strategies may include genetic techniques such as RNAi biopesticides (Murphy et al. 2016). Little information is available at the population level on the impact of insecticide sprays. Insecticides are typically targeted against specific life stages of *D. suzukii* and result in differential levels of mortality on the different life stages. Currently, calendar-based insecticide spray intervals are focused on preventing oviposition by *D. suzukii* (Cini et al. 2012; Beers et al. 2011; Bruck et al. 2011), but their impacts on populations over a larger spatial scale are unknown. Organophosphate, pyrethroid, carbamate, spinosyn, and some diamide insecticides show efficacy against *D. suzukii* adults (Bruck et al. 2011; Van Timmeren and Isaacs 2013). Residual activity of currently available insecticides is between 5 and 10 days but can be shorter due to rainfall (Van Timmeren and Isaacs 2013). There is increasing evidence that some insecticides that are active on adult *D. suzukii*, including spinosad family compounds, which, may also achieve control through mortality of egg and larval life stages (Wise et al. 2015).

Biological control agents known to attack *D. suzukii* have been identified in areas of recent pest invasion (Miller et al. 2015). However, parasitoid success appears generally lower in these regions compared to levels observed in the indigenous range of the pest (Kacsoh and Schlenke 2012; Rossi Stacconi et al. 2015; Daane et al. 2016). In North America and Europe, specialist parasitoid species are absent. Field studies indicate that natural populations of generalist species are not having a meaningful effect on populations of this pest; however, in the scope of an IPM program, a conservation biological control approach using these agents may contribute to an overall reduction in local *D. suzukii* populations (Miller et al. 2015; Rossi Stacconi et al. 2015; Wang et al. 2016).

The complex of biological control agents for *D. suzukii* includes predators and pathogens (Woltz et al. 2015); however, parasitic hymenoptera have been the primary focus of current research. Numerous parasitoid species are known to attack frugivorous drosophilids and most attack larvae or pupae in decaying fruits on the ground (Hertlein 1986; Fleury et al. 2009). Recent studies in the U.S.A. and Europe found that most resident larval drosophila parasitoids were unable to develop on *D. suzukii* (Chabert et al. 2012; Kacsoh and Schlenke 2012), but in Asia, several parasitoid species of *Asobara*, *Ganaspis*, and *Leptopilina* can attack and develop from larvae of *D. suzukii* (Kasuya et al. 2013; Mitsui et al. 2007; Nomano et al. 2015). Collection trips to South Korea in 2013 and 2014 and China in 2013 yielded parasitoid species that readily attack *D. suzukii* larvae and pupae (Daane et al. 2016).

Given the increasing availability of *D. suzukii* physiology data, the goal of this paper is to provide key insights into how physiological time can be utilized to integrate survival, development, and reproductive data from diverse environments. We demonstrate how physiological time is appropriate to describe population dynamics over the growing season. We also demonstrate how the physiological time concept breaks down during overwintering by examining how extreme temperatures cause mortality in non-acclimated *D. suzukii* at both high and low temperatures. *D. suzukii* enters reproductive diapause in November/December in parts of the U.S.A. (Wallingford et al. unpubl.), and phenotypic changes among individuals in the population can affect winter survival (Shearer et al. 2016). We focused on the latter portion of winter and spring to determine if DD accumulation could estimate female reproductive potential. Finally, we examined a cohort-level population model based on accumulation of DD utilizing daily high and low temperatures from different field sites to estimate DD for conditions within known thermal thresholds. These data were used to consider the impacts of current and possible future IPM with the cohort DD population model at the field level.

## Materials and methods

The environmental factors described below illustrate the impacts of environmental conditions within and outside of known temperature thresholds of *D. suzukii*. Additionally, we describe the role of DD accumulation for estimating sexual maturity of reproductive flies collected during the late dormant period. During late winter and early spring, there is a transition from temperatures outside of thermal thresholds to conditions falling within thermal thresholds. The *D. suzukii* population model was used to demonstrate how management practices could affect populations on a relative scale.

### Parameters for environmental conditions falling within temperature thresholds

Emiljanowicz et al. (2014) found that survival and developmental times from egg to adult on artificial food media were similar to rates found in *D. suzukii* reared on cherries (Tochen et al. 2014). Tochen et al. (2014) used fruit to determine development to the adult stage, so data on the survival of immature life stages were not collected, as this would require destructive sampling. Thus, our model incorporated mortality data from both sources, using age-specific mortality for eggs and larvae from Emiljanowicz et al. (2014) and mortality of all other age classes from Tochen et al. (2014). These data were fitted using the Gompertz (1820) function and using the two-parameter probability density function:

$${\text{F}}\left( {{\text{x}}|a, \, b} \right) \, = { \exp }\left( { - b/a\left( {{ \exp }\left( {a{\text{x}}} \right) - 1} \right)} \right),$$

where *a* is the shape and *b* is the rate. Data were fitted using the open-source statistical environment R version 3.2.2 (R Development Core Team 2015). Survival data were fitted using R packages “survival” (Therneau and Lumley 2015) and “flexsurv” (Jackson 2015). Pearson’s Chi-square was used as a goodness of fit test for survival and maternity models (Agresti 2007).

Maternity over physiological time for *D. suzukii* was plotted using data originating from temperature-dependent life table studies performed at constant temperatures on adult *D. suzukii* using cherry (Tochen et al. 2014). Calendar time was converted to DD for these experiments using the upper and lower development thresholds *T*_H_ = 30° C and *T*_L_ = 7.2° C, respectively (Tochen et al. 2014). These thresholds were used for all subsequent DD calculations. We summed all eggs laid during six periods of similar DD accumulation, i.e., 150–230, 231–310, 311–390, 391–470, 471–550, and 551–610 DD in order to represent the adult lifespan in DD. The number of eggs laid per DD was plotted over the midpoint for each range of DD. These data were fitted using the Cauchy distribution:

$${\text{F}}\left( {x_{0} |\varUpsilon } \right) \, = { 1}/\pi \varUpsilon \left( {\varUpsilon^{ 2} /\left( {{\text{x}} - x_{0} } \right) + \varUpsilon^{ 2} } \right),$$

where the parameter *x*_*0*_ is the location and *ϒ* is the scale.

### Estimation of survival outside of temperature thresholds for development

We examined survival trends of *D. suzukii* populations under cold and warm temperature extremes outside the developmental and reproductive thresholds. Populations are expected to decrease substantially after exposure to extremes (Asplen et al. 2015); however, even after extended periods of unfavorable conditions and lack of suitable reproductive hosts, *D. suzukii* are known to respond to traps, indicating persistence of populations (Dalton et al. 2011; Wiman et al. 2014). We describe the impacts of such unfavorable conditions on population structure by plotting *D. suzukii* pupal and adult survival levels at extreme low (1, 3, 5, and 7 °C; Dalton et al. 2011) and extreme high (30 °C; Tochen et al. 2014) temperatures. Survival was fitted in this case with a Gompertz distribution over calendar days because no DD are accumulated at the extremes. Currently, we lack field data to illustrate the role of such environmental conditions on populations. We do not include these parameters in the model described below as this paper focuses on seasonal population fluctuation only.

### Late dormant reproductive potential

In this analysis, we examined how warming temperatures at the onset of the growing season affect female reproductive potential of field-collected *D. suzukii.* The goal was to determine if DD accumulation could be used to estimate reproductive potential of flies, and to determine whether laboratory-generated reproduction data are supported by field observations. Collections of females were conducted using established methods and the late dormant reproductive potential of *D. suzukii* females was classified by dissection of females under magnification to determine whether mature eggs were present and if they were in the ovaries or free in the abdomen (Boulétreau 1978; King 1970; Watabe and Beppu 1977). Collections from Seattle, Washington, U.S.A. were made from March 2011 to February 2012, and collections from Corvallis, Oregon, U.S.A. were made from April 2011 to June 2013 (Table 1). Flies were collected using container traps baited with apple cider vinegar or yeast-sugar solution. Collections from Italy were made from January to April 2015 in multiple locations (Table 1). Collections in Italy utilized container traps baited with 200 ml of the liquid bait Droskidrink (Grassi et al. 2015), composed of 3 parts apple cider vinegar to 1 part red wine, with 4 g raw brown sugar dissolved into the mixture. In all sites, the total numbers of females dissected per location and date were used to calculate the percentage of females containing mature eggs. The percentage of females containing mature eggs was plotted over the midpoint for the time period in DD calculated from the daily high and low temperature using the single sine method. Temperature data originated from weather stations proximate to collection sites representing the regions where collections were made. In all regions, the relationship between accumulated DD and reproductive potential was determined with multiple regression (R Development Core Team 2015).

**Table 1:** Collection sites of late dormant female *D. suzukii* dissected in order to determine reproductive potential

Collection Site	State, Country	Latitude	Longitude	Elevation (m)	Land Use
Zambana Vecchia	Trentino, Italy	46°09′30″N	11°04′35″E	203	Commercial sweet cherry orchard
Romagnano	Trentino, Italy	46°00′26″N	11°07′2″E	190	Commercial sweet cherry orchard
Pergolese	Trentino, Italy	46°01′48″N	10°57′35″E	250	Commercial sweet cherry orchard
Riva del Garda	Trentino, Italy	45°53′35″N	10°51′15″E	77	Commercial sweet cherry orchard
Roverè della Luna	Trentino, Italy	46°15′30″N	11°10′29″E	234	Forest
Susà	Trentino, Italy	46°02′40″N	11°12′26″E	748	Forest
S. Michele all’Adige	Trentino, Italy	46°11′17″N	11°08′13″E	272	Composting area
S. Michele all’Adige	Trentino, Italy	46°11′34″N	11°08′22″E	272	Grape orchard
Mezzocorona	Trentino, Italy	46°12′40″N	11°07′28″E	213	Private garden
Trento	Trentino, Italy	46°04′03″N	11°08′11″E	252	Private garden
Trento	Trentino, Italy	46°03′15″N	11°07′25″E	195	Public garden
Trento	Trentino, Italy	46°03′59″N	11°08′12″E	242	Public garden
Viarago	Trentino, Italy	46°04′40″N	11°15′58″E	656	COMMERCIAL red raspberry field
Vigalzano	Trentino, Italy	46°04′25″N	11°14′01″E	507	Ivy hedge
Seattle	Washington, U.S.A.	47°33′48″N	122°′16′08″W	57	Unsprayed mixed small fruit and cherry fields
Corvallis	Oregon, U.S.A.	44°33′38″N	123°13′38″W	60	Unsprayed mixed small fruit and cherry fields

### Environmental population model construction

For conditions within the developmental thresholds, daily DD accumulation was used to model survival and reproduction of individual cohorts of *D. suzukii.* For each daily DD accumulation for a modeled population, the fitted DD models for survival (Gompertz) and reproduction (Cauchy) were used to determine the life table statistics: age-specific survival (*L*_*x*_); age-specific gross maternity (*M*_*x*_); and net maternity (*L*_*x*_*M*_*x*_) (Carey 1993), where age (*χ*) is the cumulative DD for each individual cohort. Thus, DD accumulation estimated variable rates of survival and reproduction on a daily basis. For each DD increment, the population size for each cohort was estimated by net maternity (*L*_*x*_*M*_*x*_). The daily sum of net maternity from all reproductive cohorts, $\mathop \sum \nolimits L_{x} M_{x}$, constituted a new cohort aged 0 DD. In order for reproduction to occur, cohorts must have achieved between 210 and 610 DD, denoting the adult stage, and temperature had to be within the reproductive thresholds (*T*_H_ = 29.3 °C and *T*_L_ = 13.4 °C) (Tochen et al. 2014). No studies have shown reproduction of *D. suzukii* outside of the range of these thresholds.

### Impacts of insecticide and genetically engineered biopesticide on *D. suzukii* populations

For the model runs, we used two of the temperature datasets originally from Wiman et al. (2014). The first was from the 2013 growing season in Salem, Oregon, U.S.A. and the second from 2013 in Parlier, California, U.S.A. Mortality factors simulating management activities were applied to select life stages for the periods outlined below. Model runs started early in the season because the population structure during the beginning of the growing season was composed of mostly adults. This timing allows us to see how pesticides targeting adult or immature life stages perform in relative terms. For California, we assume that adults colonize blueberry fields to oviposit on ripening fruit on April 1. Whereas growers would likely apply insecticides more than one time per season, for simplicity, hypothetical insecticides were applied one time at the beginning of the season. Two insecticides with different effects on specific life stages were independently input into the model to compare population-level impacts. The two compounds represented active ingredients that control both adults and immature stages of *D. suzukii* at different levels (Beers et al. 2011; Bruck et al. 2011; Van Timmeren and Isaacs 2013). Insecticide A elicited an adult mortality factor of 95 % and an immature mortality factor of 5–100 %. Insecticide B caused 60 % adult mortality and 60–95 % mortality of immature stages. These mortality factors included a range of efficiency in order to simulate reduced residual activity over time.

We compared conventional insecticide treatments with RNA interference (RNAi) as a treatment. This technology has undergone major advances as a tool for pest management. Double-stranded RNA (dsRNA) is administered to targeted insects by genetic modification of the crop, or synthesized in vitro and topically applied to host plants (Li et al. 2015). Murphy et al. (2016) described a novel dsRNA delivery system in which researchers genetically engineered yeast to produce dsRNA that knocks down genes that are predicted to be critical for *D. suzukii* fitness. The yeast biopesticide, Insecticide C, was shown to decrease larval survivorship and to reduce adult locomotor activity and reproductive output. Using these findings, we applied realistic mortality levels as highlighted by Murphy et al. (2016), assuming efficient delivery and persistence, in which *D. suzukii* egg production and egg viability was 63.2 % lower (41.5 % fewer eggs produced and 21.7 % fewer viable eggs), and 22 % of the larvae were killed for a period of 7 days. The mortality factors for each class of toxicant were applied using weather data from Parlier, California, U.S.A. for 20–30 April 2013 using these treatment scenarios.

### Parasitism impact on *D. suzukii* populations

Oregon weather data were used to illustrate the impact of the different levels of parasitism on *D. suzukii* populations. In the U.S.A. and Italy, the current suite of parasitoids attack only late larval and early pupal life stages (Rossi Stacconi et al. 2015) of *D. suzukii*, and field rates of parasitism are estimated to be around 2 % (Miller et al. 2015). In the biological control model runs, we made the simplifying assumptions that parasitism remains constant and that parasitism is attributable only to limited biological control agents based on field observations (Miller et al. 2015). For Oregon, we also assumed abundance of alternate hosts in surrounding vegetation from March 1, and that oviposition is possible as soon as the reproductive thresholds are met (Lee et al. 2015a). Collections from South Korea show parasitism rates as high as 17 % (Daane et al. 2016). For these reasons, 2 and 15 % parasitism on the late larval-early pupal life stages of *D. suzukii* were incorporated into the model on a season-long basis and compared to populations exhibiting no parasitism. These model runs would illustrate current and potential future population impact because of classical biological control using parasitoids.

## Results

### Parameters for environmental conditions falling within temperature thresholds

**Fig. 1:**
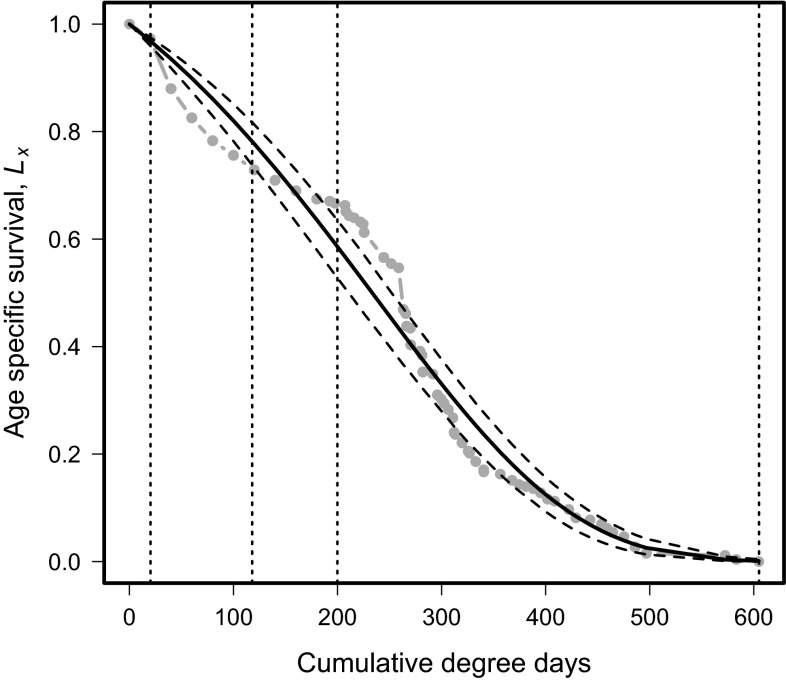
Age-specific survival, *L*_*x*_ of *Drosophila suzukii* over physiological time (degree days). Vertical dashed lines indicate transition between life stages from left to right, i.e., eggs, larvae, pupae, and adults. The *vertical dashed line* to the *right* indicates when 100 % mortality occurs. *Dashed lines* indicate 95 % confidence intervals

The Gompertz distribution with parameters (*a* = 5.3, *b* = 1.5; *χ*^2^ = 0.750; d.f. = 1, 251; *p* = 0.01 Fig. 1)) provided a good fit to survival of *D. suzukii* over DD. The DD ranges for each life stage were eggs = 0–20.27 DD; larvae = 20.28–118 DD; pupae = 118.1–199.9 DD; and adults = 200–610 DD, as determined using the fecundity data from Tochen et al. (2014). The initial immature life stages experience a comparatively low mortality rate up to 199.9 DD. Thereafter, the slope of the mortality curve increases dramatically until it reaches 400 DD, after which it flattens out. The proportion of surviving insects reaches 0 at ca. 610 DD.

**Fig. 2:**
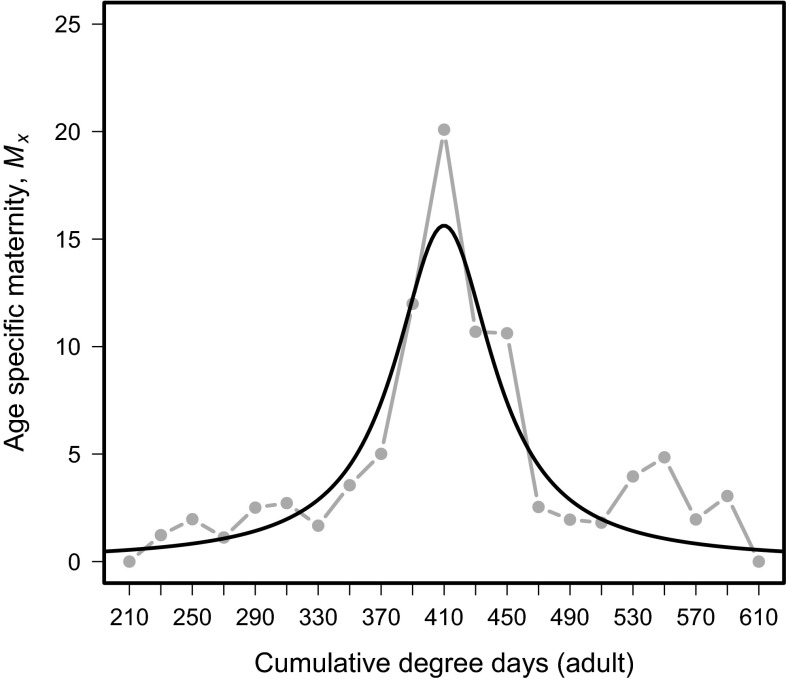
Age-specific maternity, *M*_*x*_ of adult *Drosophila suzukii* over physiological time (degree days)

Gross maternity (*M*_*x*_) for *D. suzukii* females on a DD scale was described by the Cauchy distribution with parameters (*l* = 1; *s* = 1.9; *χ*^2^ = 9.9605; d.f. = 1, 20; *p* = 0.97; Fig. 2). Using this function, egg laying initiates at approximately 210  DD and gradually increases to a maximum estimation of 15 eggs per DD at 410 DD. Egg laying subsequently decreases up to 610 DD, at which point it ceases.

### Estimation of survival outside of temperature thresholds for development

**Fig. 3:**
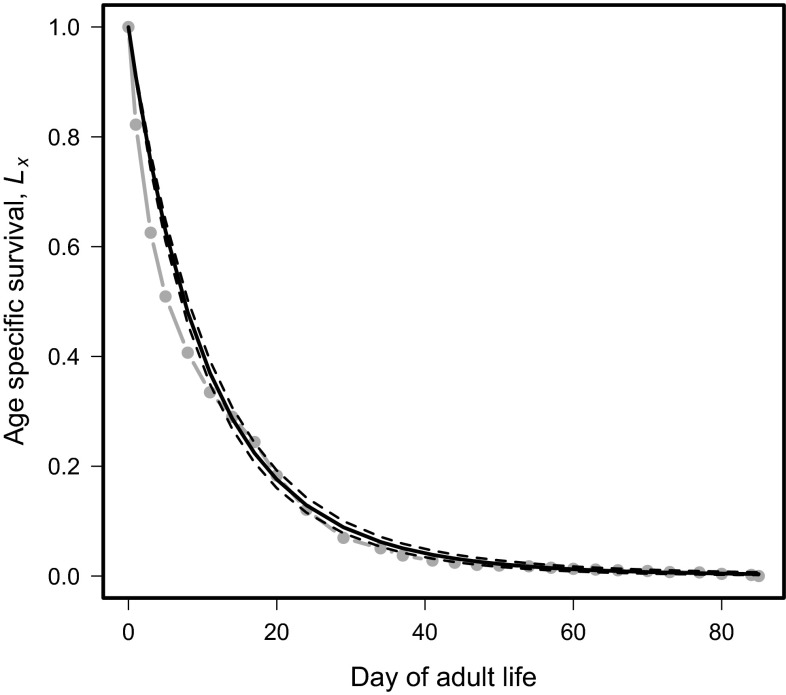
Age-specific survival, *L*_*x*_ of adult *Drosophila suzukii* over time (days) at temperatures outside of known developmental thresholds. *Dashed lines* indicate 95 % confidence intervals

Under extreme cold-temperature conditions, the adult survival data fit a Gompertz curve (*a* = −0.009, *b* = 0.09501; *χ*^2^ = 1.07; df = 1, 139; *p* value 1; Fig. 3). The curve shows high mortality levels from days 1–30, after which mortality rates become more gradual and the curve reaches the lower asymptote.

### Late dormant reproductive potential

**Fig. 4:**
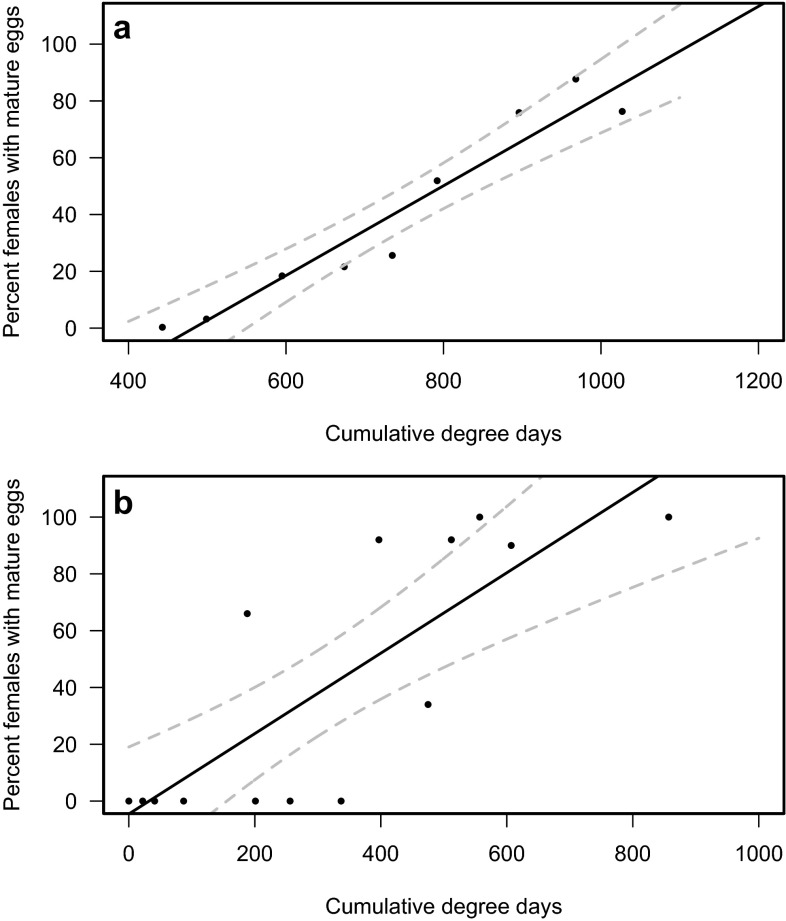
Percentage of *Drosophila suzukii* with mature eggs from January to June as determined by dissecting females under a microscope. Data were collected in Trento, Italy (**a**) during 2015. In Corvallis, Oregon, U.S.A. and Seattle, Washington, U.S.A., data were collected during 2011 through 2013 (**b**). *Dashed lines* indicate 95 % confidence intervals

The late dormant reproductive potential generally showed an increase in egg maturity levels in all regions. In Trento, Italy, the regression, *y* = 0.1579*x* − 76.24 (*R*^2^ = 0.92; *F* = 88.2; df = 1, 7; *p* < 0.001; Fig. 4a), showed an increase starting at 500 DD and reached a peak at approximately 1000 DD during the 2015 season. Data collected from the U.S.A. from 2011 through 2013 resulted in a significant fit with the function *y* = 0.14*x* − 4.55 (*R*^2^ = 0.64; *F* = 26.02; df = 1, 13; *p* < 0.001; Fig. 4b) describing the increase in reproductive maturity. Here egg maturity increased starting as early as 50 DD to a maximum of 800 DD.

### Impacts of insecticide and genetically engineered biopesticide on *D. suzukii* populations

**Fig. 5:**
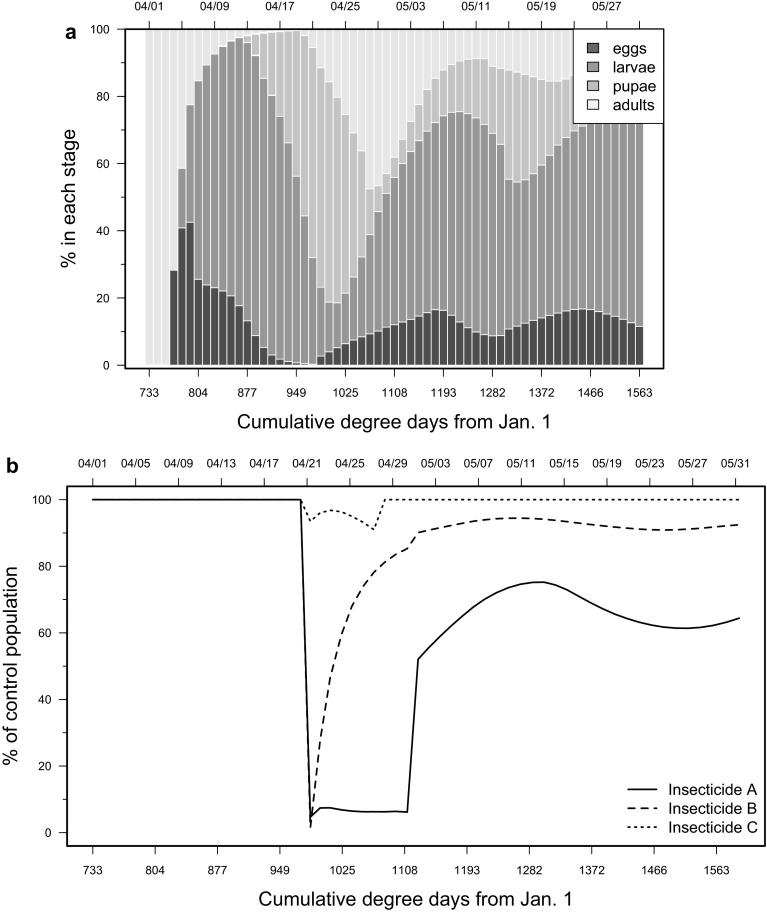
*Drosophila suzukii* population model outputs showing life stages for the control populations (**a**) and relative impacts for Insecticide A, Insecticide B, and Insecticide C compared to control (**b**). Environmental conditions from Parlier, California, U.S.A. during April 21 to May 21, 2013 were used as model inputs

Model outputs provided clear trends in comparative population decrease. The population structure of the control population (without insecticide treatment) at the beginning of the growing season in California showed that 100 % of the total populations are adults (Fig. 5a), with oviposition initiating at 750 DD. The proportion of the adult population started to decrease at this point as the frequency of other life stages increased. Adult populations fluctuated between 0.5 and 50 % up to 1563 DD or 27 May. Insecticide A initially caused a population decrease to less than 10 % of the control population with a delayed population recovery. Insecticide B resulted in an equal initial population decrease, but there was faster recovery of *D. suzukii* compared to Insecticide A. Insecticide C, the genetically engineered biopesticide, had a seven-day residual period and resulted in up to 5 % reduction in populations as compared to untreated populations (Fig. 5b). For all insecticide treatments, the model outputs showed fluctuations in *D. suzukii* pest populations, although the fluctuations were less pronounced for Insecticides B and C compared to Insecticide A. It is also clear that insecticides administered during this period resulted in a general decrease in populations of *D. suzukii* under California conditions.

### Parasitism impact on *D. suzukii* populations

**Fig. 6:**
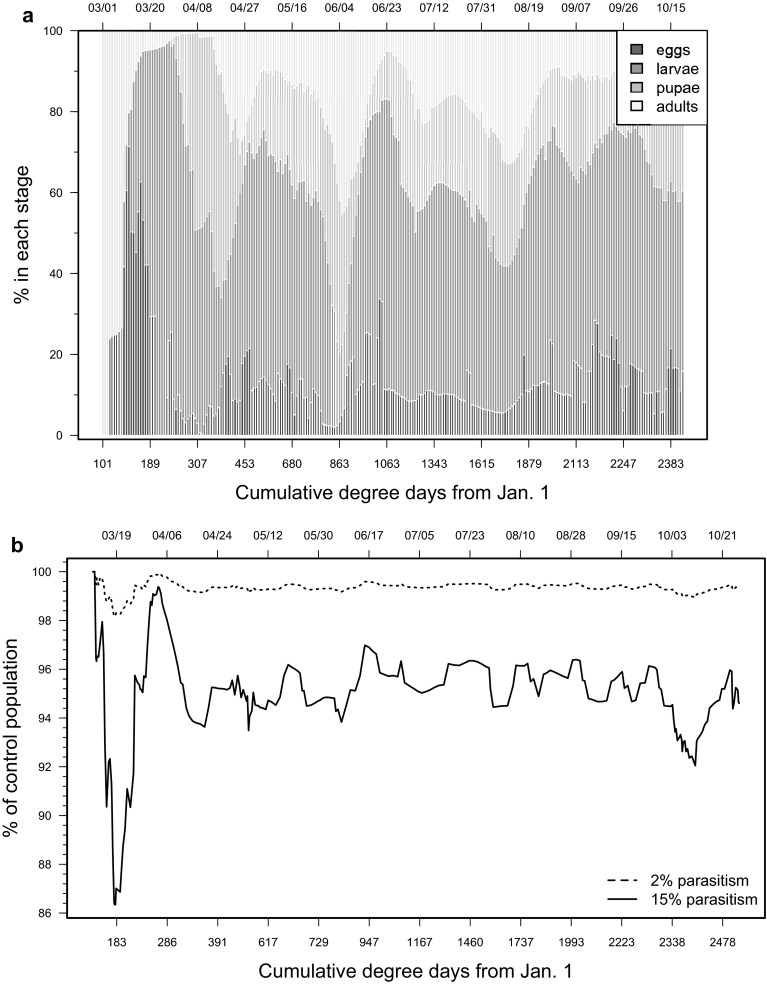
*Drosophila suzukii* population model outputs showing life stages for the control populations (**a**) and relative seasonal impact of larval parasitoids at 2 and 15 % parasitism levels compared to no suppression during 2013 in Salem, Oregon, U.S.A. (**b**)

The population structure of *D. suzukii* in Oregon initiated with 100 % of individuals in the adult stage at 101 DD (Fig. 6a). This proportion declined as oviposition started. Adult populations ranged between 2 and 45 % for the remainder of the season. In Oregon climates, the model indicates that season-long biological control can result in reductions of *D. suzukii* populations relative to populations not affected by parasitism (Fig. 6b). At the 2 % parasitism level, populations were reduced by approximately 1–2 % at the end of the growing season. The highest level of population reduction is estimated at 4 % and the lowest at 1 % compared to populations not affected by parasitism. At 15 % parasitism, *D. suzukii* populations were reduced by ca. 10 % compared with populations not affected by parasitism. The highest level of population reduction is estimated at 21 % and the lowest at 1 % compared to populations not affected by parasitism. At the 15 % parasitism level, the impact on *D. suzukii* populations is rapid but variable during the early portion of the season, with lower levels of population decrease during the latter portions of the season at the time when adult populations are elevated.

## Discussion

This research suggests that DD are very useful for estimating physiological time, and their use extends beyond phenology models. In addition, we describe the impacts of IPM strategies on early-season *D. suzukii* populations presumed to consist mainly of adults. The data presented are an alternative to the model developed by Wiman et al. (2014) to estimate populations of *D. suzukii* within environmentally known thermal thresholds. Previously, insects were forced to progress through the age-structured matrix with mandatory daily advancement without taking into consideration physiological age. An advantage of the current model structure is that it allows flies to proceed through the physiological age-structured matrix contingent on accumulation of physiological time as approximated by DD. This refinement to the model allows survival and reproduction of concurrent cohorts comprising a population to be more precisely estimated. The two functions that describe mortality and fecundity within thermal thresholds in physiological time provided good fit using standard population survival and fecundity fitting techniques. We realize that a partial or complete shift of phenotypes in response to temperature extremes (Shearer et al. 2016) is not taken into account. However, future modeling efforts will be aimed at capturing phenotypic plasticity and its implications for survival and reproduction of populations experiencing extreme environmental conditions.

We provided parameters of *D. suzukii* survival outside of thermal development thresholds, simulating a sudden transition to extreme cold. The fitted exponential function describes the survival rates of un-acclimated *D. suzukii* populations under prolonged environmental conditions. When exposed to suboptimal temperatures, un-acclimated *D. suzukii* populations clearly display high mortality rates. Theoretically, the horizontal asymptote of the fitted exponential function will not result in the extinction of winter or summer populations under these extreme conditions, and in the field, this phenomenon is observed as populations build during periods of favorable temperatures. Investigation of this observation, however, falls outside the scope of the current study where we focused on the growing season. We therefore realize that the ability to estimate population response to a wide range of changing environmental conditions will further improve population estimation of adaptive invasive species such as *D. suzukii*.

One step was made to more clearly understand how improving environmental conditions impact winter-surviving *D. suzukii* reproductive potential. Dissections of late dormant female *D. suzukii* from Oregon and Washington, U.S.A., and Trentino, Italy, displayed increasing levels of reproductive maturity as DD accumulated. Our laboratory data show that egg laying is initiated at 210 DD, whereas field increases in reproductive potential range from 50 to 800 DD. There may be various explanations for these differences, including year, microclimate, genetic variability, and trapping techniques. Although the timing of reproductive maturity with DD in these studies differed, the data nevertheless show a clear relationship between reproductive potential and physiological time. These data illustrate the importance of more suitable environmental conditions as a factor contributing to increased reproductive potential of *D. suzukii*. We realize that temperature is not the only factor contributing toward such increased reproductive potential, as the roles of humidity, alternate food sources, and host media during the late dormant period of *D. suzukii* may also need consideration (Lee et al. 2015a; Tochen et al. 2015). Our data suggest that in some regions, female sexual maturity may occur very early in the growing season before hosts become widely available. The lack of suitable fruit hosts in certain areas early in the growing season likely makes *D. suzukii* more dependent on alternate nutrient sources. These sources include pollen and nectar, which may be utilized by *D. suzukii* to increase reproductive potential and survival levels. Such resource availability may be a factor resulting in variability of our data. For these reasons, it is important in the future to describe the contribution of these different factors to *D. suzukii* population dynamics. Clear parameters should be developed to more accurately model early-season *D. suzukii* population increase.

We used a model to illustrate the importance of key periods when pest population structures can be exploited to the advantage of IPM (Thomas 1999). To the best of our current understanding of the structure of *D. suzukii* populations, adult females comprise the majority of overwintering individuals (Dalton et al. 2011; Wiman et al. 2014). Thus, early spring is a key period when only sexually maturing adults are present. Elimination of these initial adults before the population becomes established and spread among different ages would be ideal. In Parlier, California, U.S.A., a second key period exists during the summer when suboptimal hot temperatures prevail and *D. suzukii* populations decline.

Insecticide A had a longer-lasting residual against adult stages and a shorter residual against immature stages as compared with Insecticide B, which explains the strongest effect on the early-season population. Insecticide B model runs simulated compounds designed to cause mortality at all life stages of *D. suzukii* (Wise et al. 2015). These compounds are of increasing importance as the population age structure becomes more diverse. In the early season, as in our simulation, adults dominate. During latter portions of the season, larger portions of *D. suzukii* populations are expected to be in immature life stages. During such latter portions of the growing season, there should be increased focus on pesticides that target all life stages of *D. suzukii*. The use of adulticides to prevent fruit rejections due to infestation before harvest when all ages of flies are present, however, will remain a key component of IPM programs. Additional considerations include the development of *D. suzukii* insecticide protocols to minimize the development of insecticide resistance.

Genetic control using RNAi biopesticide (Insecticide C) technology with seven-day residual periods against all life stages resulted in minimal population reduction compared with untreated populations. Again, targeting of larvae makes more of a difference on structurally diverse populations. The utility of this technology to target immature life stages of *D. suzukii* may make it more effective at curatively managing pest populations compared to the adulticides during periods when immature life stages dominate the population structure. An additional advantage of such genetic pest management tools is that they can be designed to be species-specific and target *D. suzukii* only. Disadvantages currently include regulatory obstacles, wary public perception, and potential incompatibility with organic production practices.

Both levels of biological control inputted into model runs resulted in *D. suzukii* suppression. Higher levels of parasitism will probably result in concomitantly lower levels of pest pressure during the harvest period. Currently, only low levels of biological control are found in most production regions (Miller et al. 2015; Rossi Stacconi et al. 2013, 2015). Model runs indicate that levels of parasitism close to 15 % will result in significant reductions of pest populations during the early portion of the season due to the loss of a portion of the initial crop-infesting population. Biological control is only effective, however, if it can suppress pest populations as the crop ripens. Model outputs estimate lower levels of suppression during the earlier portion of the season at the 2 % level of biological control. Clearly parasitism, particularly at the higher rate, helped to destabilize the *D. suzukii* population and is an effect that may be enhanced by additional compatible control measures. Overall, these data suggest that biological control, as it currently stands, will not be effective as a standalone management tactic but will result in additive contributions to IPM programs targeting *D. suzukii*. Increased benefit will undoubtedly be gained from additional classic biological control introductions.

We believe that the refined model presented here can be used as a comparative tool for practitioners and scientists, and such models will allow for the integration and optimization of multiple IPM technologies. This approach also illustrates that IPM practitioners should take advantage of environmental conditions that create vulnerability of the pest to management activities. During more suitable summer conditions, alternative factors such as pesticide use and biological control are considered as key management techniques. We realize that manipulations of *D. suzukii* populations are not the only factors that can be used in IPM strategies. Behavioral techniques including push–pull strategies, cultural methods, and insect barriers could also contribute to sustainable management of *D. suzukii*.

In practice, IPM strategies often focus on single technologies including biological control, host plant resistance, chemical pesticides, or biopesticides. Typically, very little attention is given to the interaction or compatibility of the different technologies used and their timing (Thomas 1999). Future studies using stage-structured models such as the one presented here should be conducted to investigate these interactions. We realize that population modeling is only one approach for understanding how to manage this damaging pest. However, it can provide powerful insights into the relative performance of different tactics and combinations, and we expect that population modeling will allow rapid assessment of different integrated control programs and their expected performance under different environmental conditions.

## Author contribution

NGW, DTD, GA, AB, JCC, KMD, BG, AG, KAH, RI, AG, CI, JCL, BM, MVRS, PWS, LT, XW, and VMW conceived and designed research. NGW, JCL, and VMW conducted experiments and analyzed data. All authors contributed to writing the paper.
